# Prevalence, characteristics and measurement of somatic symptoms related to mental health in medical students: a scoping review

**DOI:** 10.1080/07853890.2023.2242781

**Published:** 2023-08-08

**Authors:** Edie L. Sperling, Jennifer M. Hulett, LeeAnne B. Sherwin, Sarah Thompson, B. Ann Bettencourt

**Affiliations:** a Sinclair School of Nursing, University of Missouri, Columbia, MO, USA; b College of Osteopathic Medicine of the Pacific-Northwest, Western University of Health Sciences, Lebanon, OR, USA; c Ellis Fischel Cancer Center, University of Missouri, Columbia, MO, USA; d Psychological Sciences, University of Missouri, Columbia, MO, USA

**Keywords:** Medical students, stress, somatization, mental health

## Abstract

**Introduction:**

Somatic symptoms related to mental health in medical students are under-researched, with nothing on the topic being published in the United States in over three decades. This scoping review is the first of its kind to explore the prevalence, type and severity of somatic symptoms induced by stress, anxiety, depression and burnout amongst medical students, with the objective of describing the significance and breadth of this issue.

**Methods:**

PRISMA-ScR guidelines were used to guide this review. A comprehensive search was performed of 22 databases, followed by bibliographic and hand searching. Inclusion criteria were published, peer-reviewed articles with a sample of medical students and at least one measure of somatic symptoms related to mental health, in English or with an English-language translation. Excluded were review, companion and editorial articles. Coding was done by an experienced coder trained in systematic review techniques. Two authors reviewed each article.

**Results:**

Twenty-nine articles met inclusion criteria, representing 16 countries, 31 schools/teaching hospitals and 9,887 medical students. The prevalence of somatic symptoms ranged from 5.7 to 80.1%, and somatic symptoms were overwhelmingly found to be significantly correlated with mental ill-health. Somatic symptoms included back pain, neck pain, headaches, sleep disturbances and functional gastrointestinal disorders. Eleven different outcome measures were used, with varying degrees of validity and reliability, which were compared and assessed.

**Conclusions:**

Somatic symptoms appear strongly correlated with mental ill-health in medical students, and are likely highly prevalent. This review highlights the need for further research on somatic symptoms of mental ill-health in medical students, particularly in the United States, and the addition of larger, multi-institutional cohorts to expand our understanding of prevalence, incidence and inciting factors of somatic symptoms. Longitudinal studies tracking somatic symptoms’ effect on career trajectory and professional burnout levels are also needed. Finally, future research should explore interventions for reducing physical symptom burden in medical students.

## KEY MESSAGES

- This scoping review is the first of its kind to explore the breadth and depth of knowledge on the presence, prevalence, type and severity of somatic symptoms related to stress experienced by medical students across the globe, and if or how physical symptoms of stress have been addressed thus far.
- Medical students are known to have chronically high levels of stress, but somatic symptoms of stress are not well researched in this population, particularly in the United States, where no research has been done on this topic in over three decades.
- This scoping review finds that across many different countries, medical students consistently report high rates of physical symptoms, including musculoskeletal pain and gastrointestinal disorders, which are highly correlated with stress and other mental health conditions.
- This review provides the first initial assessment of the outcome measures used for somatic symptoms related to mental health.
- Further research on the impact of physical symptoms in medical students, and how this might relate to medical students’ mental health and eventual career burnout, is warranted.

## Introduction

The chronic stress in healthcare professionals has been worsening for many years [1], with recent estimates that well over half of clinicians report acute stress and depression [2,3]. Rates of depression, substance use disorder and suicide are higher in healthcare professionals than in any other profession [3], as are burnout, job turnover and complete career change [4,5], with more than a third reporting intent to leave healthcare within the next year [6,7]. Although research indicates that healthcare professionals are at increased risk of stroke, cancer, hypertension, cardiovascular disease, metabolic disorder and Type II diabetes mellitus [3], and that physical symptoms predict leaving a career in healthcare [8,9], research is limited on the potential sequelae of healthcare professionals’ mental ill-health, including physical symptoms and mental health-related illness [3], and how these might affect burnout and career attrition in healthcare.

The biopsychosocial model advances that longstanding stress causes psychological and physiological changes that can lead to chronic physical diseases [10]. Physical symptoms of stress and mental health-related illnesses cause considerably reduced quality of life, increased symptom burden and increased healthcare utilization and costs in the United States [10–12]. Psychological sequelae commonly include anxiety, depression and burnout [2–5], with burnout being specifically defined as job-related emotional exhaustion, cynicism and reduced personal efficacy [5]. There are four major pathways by which mental ill-health is thought to lead to illness: (1) a direct pathway through physiological changes in the sympathetic–adrenomedullary and hypothalamic–pituitary–adrenocortical axes due to chronic stress [13–16]; (2) through health habits, for example, depression increasing the risk for substance use disorders [16]; (3) through psychosocial factors, for example, discrimination based on race or gender, which has been shown to lead to chronically elevated stress levels and mental ill-health [17,18] and (4) through health-seeking behaviors such as visiting a healthcare provider when ill [16]. Understanding if and how chronic stress progresses to mental ill-health and then physical ill-health in healthcare workers, and the factors that may mediate this relationship, could lead to designing both individual and systemic interventions to reduce symptom burden, improve individuals’ quality of life and decrease turnover and attrition of healthcare professionals.

High rates of mental ill-health amongst healthcare professionals are hypothesized to have their origins in healthcare schooling [19], and healthcare students’ mental health is infamously poor. Medical students demonstrate significantly elevated levels of stress and depression [20–28]. High levels of stress in medical students globally have been shown to correlate with physical symptoms such as gastrointestinal disorders [29–31], musculoskeletal pain [30–37] and delayed antibody production [38], but little research on mental health-related physical symptoms amongst healthcare students has been published in the United States. There is little research on whether physical symptoms of stress eventually progress to chronic psychological or physical disease states at some point during healthcare training or professional life, or whether physical symptoms contribute to occupational burnout or a decision to leave the healthcare field. Coping strategies and personality traits may be protective, or interventions such as mindfulness may help, but further research is needed. This scoping review is the first of its kind to explore the breadth and depth of knowledge on the presence, prevalence, type and severity of somatic symptoms related to mental health experienced by medical students across the globe, and if or how physical symptoms have been addressed thus far via prevention or treatment. This gives us a sense of medical student somatic symptomology internationally, suggests avenues for future research and illuminates the need for research on mental health-related somatic symptoms in U.S. medical students.

While conducting a preliminary review of the literature, it also became apparent that there are a variety of instruments designed to assess self-reported mental health-related somatic symptoms. Therefore, this review also provides the first comparison of the validity and reliability of mental health-related somatic symptom outcome measures that have been used in the literature on medical students. The guiding questions for this scoping review were (1) what is the prevalence of somatic symptoms reported in the literature for medical students? (2) What are the types, characteristics and frequencies of the different mental health-related somatic symptoms or disorders reported in medical students? (3) What are the different outcome measures of mental health-related somatic symptomology that have been used with medical students and which are most valid and reliable? (4) Is there support for any approaches to predict, prevent or treat somatic symptoms in medical students?

## Methods

This scoping review followed the Preferred Reporting Items for Systematic reviews and Meta-Analyses extension for scoping reviews (PRISMA-ScR) guidelines [39]. The Arksey and O’Malley [40] framework was used to identify the research question, guide the search for relevant studies, select studies, extract data and summarize results.

### Eligibility

Inclusion criteria included all published, peer-reviewed, English language research between database inception and 9 September 2022, to obtain a complete collection of potentially eligible articles. The sample population was required to be medical students, or to report data separately if inclusive of more than one population (e.g. medical students and dental students). Articles must have included at least one measure of mental health-related somatic symptoms, or alternately, at least one psychological measure statistically analyzed with at least one physical measure to describe somatic symptoms; for example, if anxiety and back pain were measured and then correlated, the article was eligible. The article was required to be a primary study or a report of a primary study; reviews and editorials were excluded.

### Search strategies

A research librarian was consulted to maximize the quality of the search. English language filters were used. Databases were searched including Academic Search Elite; Alt Healthwatch; CINAHL; eBook Collection; ERIC; Funk & Wagnalls New World Encyclopedia; Health Source – Consumer Edition; Health Source – Nursing/Academic Edition; Library, Information Science & Technology Abstracts; MAS Ultra – School Edition; Primary Search; OpenDissertations; APA PsycInfo; MAS Reference eBook Collection; Primary Search Reference eBook Collection; MEDLINE; EBSCO Discovery, in the categories Complementary & Alternative Medicine, Education, Health & Medicine, Nursing & Allied Health, Nutrition & Dietetics, Pharmacy & Pharmacology, Physical Therapy & Occupational Therapy, Psychology, Public Health, Science, and Veterinary Medicine; PubMed; Trip; Web of Science; Education Full Text; and Education Index Retrospective (available titles between 1923 and 1983). Publication Finder was used to search in two highly relevant journals: Psychotherapy and Mental health-related somatics, and the Journal of Mental health-related somatic Research.

A structured search strategy was used, beginning with Boolean terms. If needed, because of an extensive number of results, these terms were then refined to MeSH terms. MeSH search terms included ‘mental health-related somatic symptoms’, ‘unexplained medical symptoms’, ‘psychophysiological disorders’, ‘psychobiological disorders’, ‘somatization’, ‘somatization’ (British spelling), ‘somatic distress’ and ‘somatoform disorder’ along with ‘medical students’. The number of duplicates, irrelevant articles, non-English language articles without translations and books were recorded. A bibliographic search was done on 10 relevant articles [35,41–48]. The articles from the databases were compared to see if there were frequently occurring journal names, which were then hand-searched; these were the *Education in Medicine Journal*, *Academic Medicine* and the *Journal of Psychosomatic Research*. Gray literature was not searched due to lack of peer-review.

### Selection process

**Figure 1.**
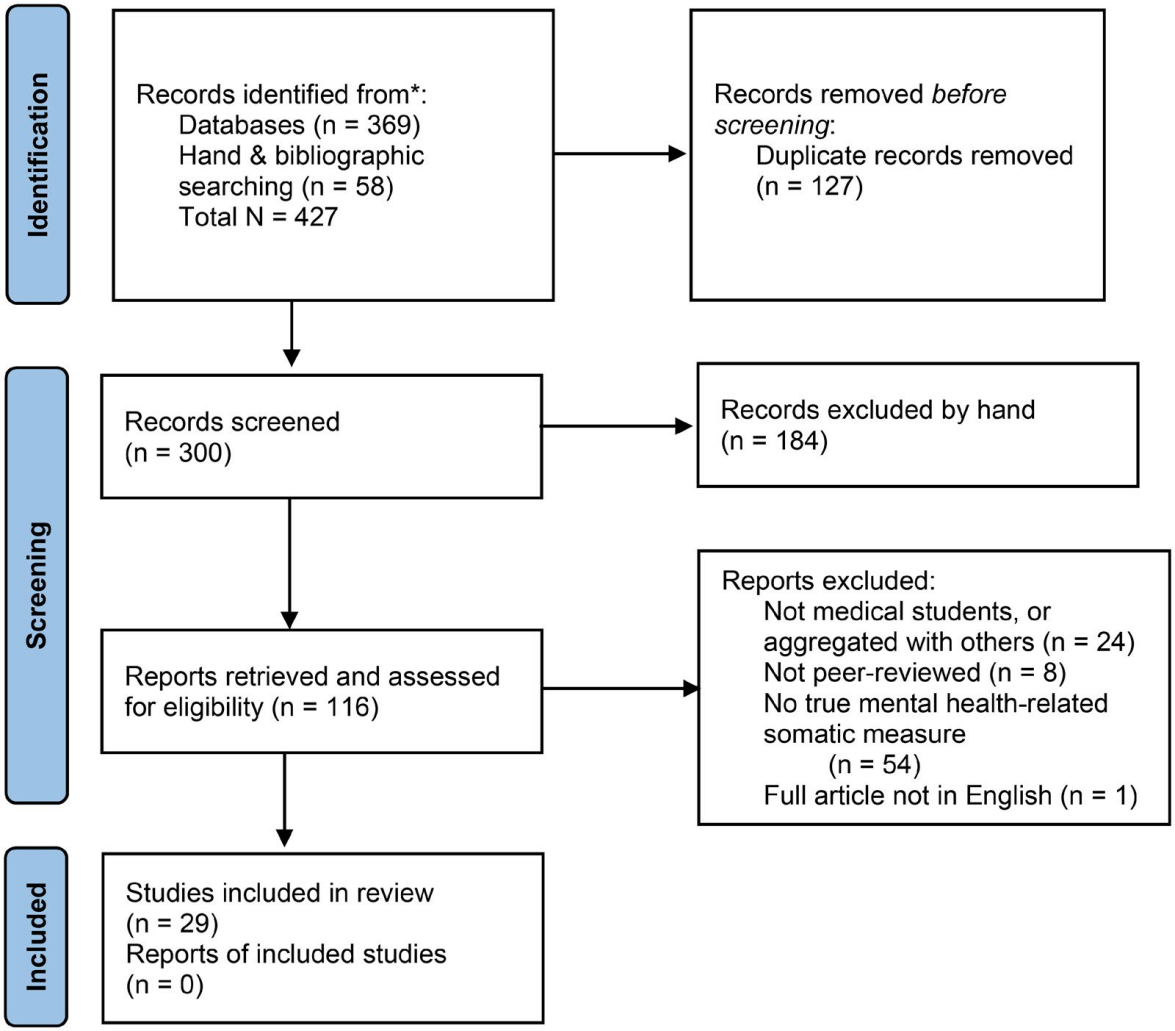
PRISMA-ScR flow diagram for study selection and screening. *From:* Tricco et al. [39].

As shown in Figure 1, 427 citations were screened (*n* = 369 from databases and *n* = 58 from bibliographic and hand searching) and 127 duplicates were removed. Records were then hand-screened by title, and 184 were removed. The remaining 116 reports were retrieved and assessed for eligibility and 87 were excluded: 21 did not have a separate data on a medical student sample, six were not a peer-reviewed primary study and 54 did not assess true mental health-related somatic issues. The final total for inclusion was 29 articles. Two authors evaluated each article to determine that it met inclusion criteria.

### Data collection process

Zotero and Excel were used to manage data, and Excel was used to track the search location, date, terms and results. A comprehensive codebook was developed per guidelines in Cooper [49]. A coder trained in systematic review methods extracted data from eligible studies. Categories of coded variables included study identification, date of coding, research design, funding, source of article, theoretical framework, population features (program type, location, mean age, gender, race, ethnicity, sample size, response rate of surveys), outcomes (name, self-reported or physiologic) and results (frequencies, means, standard deviations, confidence intervals, etc.).

### Data analysis

Quality assessment was performed by comprehensively evaluating sources of bias (see section ‘Bias’). For correlation statistics seen in Results, IBM SPSS Statistics version 29 was used.

## Results

### Study selection

Of the 29 studies included in the review, five focused on musculoskeletal symptoms, one on menstrual symptoms, two on digestive issues, one on cardiovascular symptoms and 21 on general or all symptoms caused by mental ill-health (Table 1).

**Table 1.** Study characteristics and results on mental health-related somatic symptoms in medical students.

Article	Design	Objectives	Participant characteristics	Primary outcomes measures & results
Adhikari et al. [50]	Cross-sectional descriptive	Prevalence of depression, somatic symptoms, anxiety syndromes, eating disorders, suicidal ideation, thoughts of dropping out of medical school and marijuana use in medical students at KIST Medical College and Teaching Hospital, Nepal.	*n* = 343; Age: not reported; 49.0% female	PHQ-9, PHQ-15*; Psychological variable(s): depression, anxiety, panic syndrome, eating disorders, suicidality; Somatic symptoms: 22.4% (18–26.9) medium to high severity; Females reported more somatic symptoms (30.4 vs. 14.9%; Chi-sq 11.829, *p* = .001) with menstrual symptoms, then headaches; males reported headaches first then ‘pain in extremities’. Other reported symptoms: indigestion, altered bowel, SOB, palpitations, fainting, dizzy, chest pain, sex problems, back pain, and stomach pain.
Algarni et al. [32]	Cross-sectional descriptive	Prevalence of neck, shoulder, low-back musculoskeletal pain (MSP) and associated factors in medical students at College of Medicine at King Saud University and King Saud Bin Abdulaziz University for Health Sciences, Saudi Arabia.	*n* = 469; Mean age: 21.4 (1.3); 60.6% female	SNQ; Psychological variable(s): depression; Musculoskeletal pain (MSP) is associated with depression related to medical school (OR 2.93, CI 1.73–4.98, *p* < .001) and other mental health-related somatic symptoms (OR 2.93, CI 1.37–5.27, *p* = .004).
Alshagga et al. [33]	Cross-sectional descriptive	Prevalence, location, and factors associated with MSP among medical students in a private Malaysian medical college.	*n* = 232; Mean age: 20.6 (2.2); 72.9% female	SNQ; Psychological variable(s): stress *via* year in school; Higher MSP in past week in clinical students (OR 2.0, CI 1.15–3.67, *p* = .015) those with history of physical trauma (OR 2.6, CI 1.22–5.29, *p* = .011) family history of MSP (OR 2.6, CI 1.22–5.29, *p* = .011), and increased BMI (OR = 1.1, 95% CI 1.0–1.1, *p* = 0.03). Higher in past year for same populations.
Behera et al. [34]	Cross-sectional descriptive	Prevalence of neck pain and the associated factors among medical students of a premier medical college in India.	*n* = 331; Mean age: 20.5 (1.7); 33.2% female	Web-based questionnaire developed using the Kobo Toolbox (Harvard Humanitarian Initiative); Psychological variable(s): stress; 2.9× greater odds of neck pain in 3rd and 4th-year students than 1st-year students. Students who attributed neck pain to academic stress had 3.5× greater odds of having neck pain (*p* < .001).
Bolatov et al. [51]	Cross-sectional descriptive	Burnout and associated factors in a sample of medical students from Astana Medical University, Kazakhstan.	*n* = 736; Mean age: 20.3 (2.74); 75.0% female	OLBI-S, CBI, GAD-7, PHQ-9; Psychological variable(s): depression, anxiety, burnout, suicidality; Having academic burnout was associated with mental health-related somatic conditions including headache (*p* < .001), tiredness (*p* < .001), and sleep disturbance (*p* < .001). Burnout levels correlate with CBI-S and OLBI-S mental health-related somatic indicators (*r* = 0.313–0.624, *p* < 0.001).
Chinawa et al. [52]	Cross-sectional descriptive	Presence of somatization in medical students at two teaching hospitals in Nigeria.	*n* = 385; Mean age: 23.55 (3.33); % female not reported	ESS*; Psychological variable(s): general/any; Prevalence of mental health-related somatic disorder: 14.3% overall (males 14.2% and females 14.4%). 11.4% had head features while 7.8% had body features of mental health-related somatic disorder.
Clarvit [53]	Cross-sectional descriptive	Prevalence of oligomenorrhea. amenorrhea, dysmenorrhea, and other during menses in a group of medical students at Albert Einstein College of Medicine, USA.	*n* = 159; Mean age: 26.8; 100% female	Questionnaire, unspecified; Psychological variable(s): stress; Prevalence of oligomenorrhea 8.4% during and 10.9% prior to medical school; amenorrhea 1.9 and 2.6% respectively; dysmenorrhea 73.2 and 71.1% respectively. No sig. correlations with medical school stressors.
Diebig et al. [42]	Longitudinal	Mediating role of learning family conflicts in medical students in two cohorts at one university in Germany.	*n* = 128; Mean age: 20.7 (3.47); 70.0% female	COPSOQ, PHQ-15*; Psychological variable(s): familial conflict; Long-term commuting is associated with poor health and somatic symptoms. Commuting strain predicted somatic symptoms. Conflicts between learning and family responsibilities affected the relationship between commuting strain and somatic symptoms (indirect effect estimate between = 0.13, SE = 0.05, 95% CI [0.05; ∞), Evidence Ratio = 250.57).
Dighriri et al. [35]	Cross-sectional descriptive	Prevalence of neck, shoulder, and lowback MSP and associated factors associated among medical students at Jizan University, Saudi Arabia.	*n* = 440; Mean age: 22.4 (1.6); 50.0% female	Questionnaire, unspecified; Psychological variable(s): stress, depression; History of trauma (OR = 2.59; 95% CI: 1.54–5.64) and history of depression (OR = 2.95, 95% CI: 1.54–5.64) were significantly correlated with MSP. Participants with history of trauma had a 2.7x higher likelihood of reporting MSP (OR = 2.70; 95% CI: 1.36–5.36). Depressive symptoms associated with studying resulted in 2x higher chance of MSP (OR = 1.94; 95% CI: 1.03–3.66). Students with mental health-related somatic symptoms had 3x greater likelihood MSP (OR = 2.98; 95% CI: 1.71–5.18).
El-Gilany et al. [54]	Cross-sectional descriptive	Find the prevalence of mental disorders and associated factors among medical students at Mansoura University, Egypt.	*n* = 900; Age: *n* = 491 under age 20, *n* = 409 age 20 or above; 53.2% female	SCL-90-R*; Psychological variable(s): depression, OCD, interpersonal sensitivity; Prevalence of somatization 21.7%. Being younger than 20 years old, a man, preclinical, living outside the campus, belonging to families of rural residence, and very low or low social class were independent predictors of somatization with AORs of 1.8, 1.6, 1.5, 8.3, 1.8, and 2.5 respectively.
Feussner et al. [37]	Cross-sectional descriptive	Examine prevalence of somatization and associated resilience factors in a sample of medical and dental students at a university in Germany.	*n* = 142; Mean age: 23.21 (3.44); 70.4% female	BDI-II, SOMS-2*, NEOFFI; Psychological variable(s): depression, resilience; A critical somatization index of 7 points (minimum) was found in 50.7% (X2[df = 1] = 4.558; *p* = .033.) 1.4% of the students fulfilled the ICD-10 and DSM-IV criteria for somatization disorder.
Fino et al. [48]	Longitudinal	Examine if trait mindfulness mitigates effects of stress, mental health-related somatic symptom burden, and sleep-wake quality in medical students at the Medical School of University of Bologna, Italy.	*n* = 305; Mean age: 20.47 (1.9); 50.4% female	STAI-Y, FFMQ, PSS, PPS*, MSQ; Psychological variable(s): stress, trait mindfulness; Total prevalence of mental health-related somatic symptoms = 19.81%. Trait anxiety was strongly associated with stress, mental health-related somatic symptoms, and sleep quality at the beginning and end of the first trimester. Mental health-related somatic symptoms also significantly increased (*Z* = −3511, *p* < .0001).
Gallas et al. [29]	Cross-sectional descriptive	Evaluate prevalence of FGIDs and associated risk factors in medical students at the Faculty of Medicine of Monastir, Tunisia.	*n* = 343; Mean age: 30.2 (0.8); 68.5% female	Rome III, Mental health-related somatic symptoms checklist*, HAD; Psychological variable(s):depression, anxiety; Probable or definite anxiety was an risk factor for functional gastrointestinal disorders (FGIDs) (OR = 2.5, 95% CI= 1.1–5.8). Probable or definite anxiety correlated to somatic symptoms (OR = 2.5, 95% CI= 1.1–5.8). Anxiety resulted in 2x greater risk of having a FGID (OR= 2.5; 95% CI: 1.1–5.8).
Glaser et al. [38]	Longitudinal	Assess the effect of academic stress on the ability to generate an immune response, in medical students at Ohio State University, USA.	*n* = 48; Mean age: 23.31 (SEM 0.20); 47.9% female	SSS-8*, PSS, biomarkers; Psychological variable(s): stress, anxiety, social support; Seroconverts from the first vaccine were shown to be significantly less anxious than those who seroconverted later (*F*(l,44) = 6.28, *p* < .02). PSS: there were significant differences between antibody positive and antibody negative groups (*F*(l,43) = 4.22, *p* < .05). Social support was significantly correlated with antibodies to HBsAg and blastogenic response to SAg for the third injection (*t* = 2.21, *p* < .05).
Goweda et al. [55]	Cross-sectional descriptive	Screen for somatic symptom disorder (SSD) among medical students at Faculty of medicine, Umm Al-Qura University, Saudi Arabia.	*n* = 374; Mean age: not reported; 44.9% female	SSD diagnostic criteria; Psychological variable(s): stress; 20.3% had very high risk for somatic symptom disorder (SSD), 18.7% had high risk, 19.8% medium risk, 24.6% low risk, 16.6% had none to minimal risk. Prevalence of SSD estimated at 39%. Females had higher risk than males (*p* = .002). Most commonly reported symptoms were feeling tired/having low energy, poor sleep, and stomach/bowel issues.
Ivashchenko et al. [56]	Cross-sectional descriptive	Investigate changes of state anxiety and autonomic symptoms among medical students at I.M. Sechenov Moscow State Medical University, Russia.	*n* = 54; Mean age: not reported; 51.85% female	STAI, VASQ*; Psychological variable(s): state anxiety; Correlation between autonomic symptoms and gender was found one day before exams (*r* = 0.46; *p* = .003) and after exams (*r* = 0.901; *p* < .001), but not two weeks before exams.
Lloyd and Gartrell [57]	Cross-sectional descriptive	Examine presence of psychiatric symptomatology in a sample of medical students at the University of Texas Medical School at Houston, USA.	*n* = 285; Mean age: 25.8; 36.5% female	HSCL; Psychological variable(s): depression, anxiety, OCD; Medical students were two SDs > than general population on the Interpersonal Sensitivity and Obsessive-Compulsive Disorder (OCD) symptom subscales. Mean score on the Depression and Anxiety subscales was one standard deviation greater. Symptoms highest in 2nd year med students. Females had overall higher scores (*t* = 3.37, *p* < .001). From Somatization subscale, headaches (*t* = 4.82, *p* < .01) and loss of sexual interest/pleasure (3.30, *p* < .01), were sig different between genders and higher in females.
Mosley et al. [58]	Cross-sectional descriptive	Prevalence and correlations between stress, coping strategies, depression, and somatic symptoms in medical students at University of Mississippi School of Medicine, USA.	*n* = 69; Mean age: 26; 32.0% female	MEHS-R, CSI, CESD-R, WPSI*; Psychological variable(s): stress, depression, coping strategies; 57% had high levels of somatic distress (mean = 0.66 (.42)). 23% met levels for clinical depression (mean = 11.48 (7.13)). Stress frequency was correlated with depression (.54, *p* < .001) and somatic symptoms (0.71, *p* < .001). Stress intensity was also correlated with somatic symptoms (0.31, *p* < .01).
Oró et al. [43]	Quasi-experimental repeated measures	Evaluate and compare effects of a mindfulness-based program in medical students at a school in Spain.	*n* = 143; Mean age: 20.28 (1.54); 73.4% female	SLC-90-R*, PSS, MBI-SS; Psychological variable(s): stress, burnout, mindfulness; Somatization decreased after a mindfulness program in the intervention group compared to the control group (*F*(1, 141) = 6.22; *p* = 0.014), as did stress (*F*(1, 141) = 8.23; *p* = 0.005). Burnout not significantly affected.
Pikó [59]	Cross-sectional descriptive, first phase of longitudinal cohort study	Describe frequency of common mental health-related somatic symptoms and variations in self-assessed health status in medical students at Albert Szent-Cyorgyi Medical University, Hungary.	*n* = 691; Age range: 18–31; 60.6% female	SPHQ; Psychological variable(s): stress; Men: backache (9.2% often, 15.8% sometimes), sleeping problems (5.5% often, 13.2% sometimes) and chronic fatigue (3.3% often, 10.7% sometimes). Female: Chronic fatigue (3.3% often, 16.9% sometimes), backache, tension headaches, and poor sleep. Women more likely than men to have experienced mental health-related somatic symptoms (*p* < .0001), and prevalence of tension headaches and chronic fatigue were significantly higher in women (*p* < 0.0001).
Ruzhenkova et al. [47]	Cross-sectional descriptive	Development of differentiated approaches to psychopharmacothe-rapy in autonomic dysfunction in medical students at Belgorod State University Medical Institute, Russia.	*n* = 166; Mean age: 18 (0.9); 77.1% female	PSS, Diagnostic research criteria ICD-10 for SAD*; Psychological variable(s): stress, depression, anxiety; 80.1% had symptoms of autonomic instability. 9% reached clinical levels of somatoform autonomic dysfunction (SAD), 3.6% were subclinical. Symptoms of autonomic instability in students with SAD were particularly severe during exams. Prevalence of symptoms of SAD: tachycardia 86%, palpitations 37%, shortness of breath 57%, difficulty breathing 33%, non-satisfaction with inspiration 43%, muscular tension 52.3%, muscular tremors 67%, rapid urination 19%, headaches 67%, anxiety 90%, difficult falling asleep 71%, and unpleasant dreams 33%. Anxiety present in 86%, 62% reached clinical levels.
Scheuch et al. [44]	Quasi-experimental repeated measures	Explore changes to physiological and biochemical parameters under mental load in medical students at Karl-Marx-University of Leipzig, Germany.	*n* = 64; Mean age: 24.3 exp group; 43.8% female	Immunological parameters, physical and mental task performance, max ergometry, mental health-related somatic complaints; Psychological variable(s): stress (‘mental load’); No significant changes in leucocytes, eosinophil granulocytes, lymphocytes, or cortisol pre/post exams. Delayed reactions improved after exams (5.2% (3) to 4.4 (3), *p* < .001) as did false reactions (2.3% (2) to 1.4 (2), *p* < .001). Trends in somatic complaints went up (2.3 (3.2) to 4.1 (3.7)) and psychological complaints went down (3.8 (3.6) to 1.8 (2.5)) pre/post exams.
Sekas and Wile [49]	Cross-sectional descriptive	Determine prevalence and incidence of stress-associated illnesses and possible sources of stress among students enrolled in MD, PhD, or MD-PhD programs at Case Western Reserve University, USA.	*n* = 334; Mean age: not reported; 3.0% female	Frequency of stress-related illnesses, stress questionnaire unspecified; Psychological variable(s): stress; Reported frequency of stress-related illnesses decreased from before medical school to during medical school (according to students’ memory). In men: episodes of depression 50% prior, 18.8% during, and insomnia 24.5% prior, 13.8% during. In women: depression 72.7% prior, 23.6 during, headaches 45.5% prior, 13.6% during, insomnia 30% prior, 15.5% during.
Smith et al. [36]	Cross-sectional descriptive	Determine prevalence and distribution of MSP among Chinese medical students at a university teaching hospital in Mainland China.	*n* = 207; Mean age: not reported; % female not reported	SNQ; Psychological variable(s): stress (‘mental pressure’); 67.6% had MSP in any body site in past 1 year, 46.9% in past week, 31.9% currently. Mental pressure was significantly correlated with LBP (OR 2.9, CI 1.4–5.9, *p* = .0030).
Sohrabi et al. [60]	Cross-sectional descriptive	Explore prevalence of probable mental disorders during the internship period of medical students at Shahid Beheshti University of Medical Sciences, Iran.	*n* = 404; Mean age: 24.7 (2.01); 53.8% female	SCL-90-R*; Psychological variable(s): depression, anxiety, OCD, interpersonal sensitivity, hostility, paranoia, psychoticism; The frequency of somatization was 16.3%. Depression was 16.8% and anxiety 18.8%.
Tan et al. [30]	Cross-sectional descriptive	Determine the prevalence of IBS and symptom subgroups in students at a medical school in Malaysia.	*n* = 533; Mean age: 22 (1.8); 57.0% female	Rome I, psychological and somatic questionnaire unspecified; Psychological variable(s): depression, anxiety; The psychological and mental health-related somatic symptoms of anxiety (OR = 2.0, CI = 1.1,3.5, *p* = 0.02), depression (OR = 2.1, CI = 1.3,3.7, *p* = 0.002), insomnia (OR = 2.3, CI = 1.3,4.1, *p* = 0.006), headache (OR = 1.7, CI = 1.0,2.8, *p* = 0.04), and backache OR = 2.0, CI = 1.2,3.4, (*p* = 0.006) were all more common in the participants who had IBS.
Tang et al. [45]	Cross-sectional descriptive	Examine associations of suicidality with psychological distress, somatic symptoms, and stressors in medical students at a large, research-intensive institution, China.	*n* = 662; Mean age: 19.85 (1.6); 59.4% female	Three Qs from the Chinese NCS; K6, PHQ-15*, ASLEC; Psychological variable(s): stress, suicidality; Prevalence of somatic symptoms: 5.7% medium or high (57.9% minimal, 36.4% low, 4.3% medium, and 1.4% high). General pain/fatigue was correlated with suicidal ideation (OR 6.11, 95% CI 2.99–12.48, *p* < 0.001) and planning suicide (OR 18.83, 95% CI 2.75–128.89, *p* = 0.003).
Vitaliano et al. [46]	Cross-sectional descriptive; two cohorts	Examine distribution and pattern of psychological distress in first-year medical students at the beginning and end of the year at the University of Washington Medical School, USA.	*n* = 175 per cohort; Mean age: 25.0 (3.2) male; 26.7 (4.1) female; 37% female	SCAS, BDI, LES, FTAS*, AES, biomarkers, WCCL, SNL; Psychological variable(s):stress, depression; Increased depression during the first year of medical school in men (*t*(194) = −3.42, *p* < .001) and women (*t*(107) = −3.38, *p* < .001). Stress also increased in men (*t*(194) = −5.13, *p* < .001) and women (*t*(107) = −4.07, *p* < .001). Problem-focused coping, and seeking of social support, resulted in less distress.
Wege et al. [61]	Cross-sectional descriptive; two cohorts	Discover prevalence of common mental disorders and the use of psychotropic substances in medical students at the University of Dusseldorf, Germany.	*n* = 590; Mean age: 21.13 (3.91); ∼70% female over two cohorts	PHQ*; Psychological variable(s): depression, anxiety, panic disorders, anger; Total prevalence of moderate to high somatization 15.7%, with significantly greater rates in women (19.9%) vs. men (5.2%, *p* < 0.005). Prevalence of major depression 4.7%, prevalence of ‘other depressive symptoms’ (subthreshold) 5.8%, panic disorders 4.4%, ‘other anxiety disorders’ 1.9%.

AES: Anger Expression Scale; ASLEC: Adolescent Self-Rating Life Events Checklist; BDI-II: Becks Depression Inventory II; CBI-S: Copenhagen Burnout Inventory for college students; CEDS-R: Center for Epidemiological Depression Scale-Revised; CSI: Coping Strategies Inventory; ESS: Enugu Somatization Scale; FTAS: Framingham Type A Behavior Pattern Scale; FFMQ: Five Facet Mindfulness Questionnaire; FGID: Functional gastrointestinal disorders; GAD-7: Generalized Anxiety Disorder; HSCL: Hopkins Symptom Checklist; HAD: Hospital Anxiety and Depression Scale; IBS: Irritable Bowel Syndrome; K6: Brief Kessler-6; LES: Life Experiences Survey; LBP: low back pain; MBI-SS: Maslach Burnout Inventory-Student Survey; MEHS-R: Medical Education Hassles Scale-Revised; MSP: Musculoskeletal pain; MSQ: Mini Sleep Questionnaire; Depression Scale; NCS: National Comorbidity Survey; NEOFFI: NEO-Five-Factor-Inventory for personality self-assessment; OBI-S: Oldenburg Burnout Inventory for college students; PHQ: Patient Health Questionnaire; PHQ-9: depression; PHQ-15: for somatization; PSP: Mental health-related somatic Problems Scale; PSS: Perceived Stress Scale; Rome III: questionnaire on functional gastrointestinal disorders; SAD: Somatoform autonomic dysfunction; SCAS: The Symptom Checklist Anxiety Scale; SCL-90-R: Symptoms Checklist 90 Revised; SNL: The Social Network List; SNQ: Standardized Nordic Questionnaire; SOMS-2: Screening for Somatoform Disorders; SPHQ: Self-perceived health questionnaire; SRQ-20: Self-Reporting Questionnaire-20; SSS-8: Somatic Symptom Scale-8; STAI-Y: State Trait Anxiety Inventory; VASQ: Veins Autonomic Symptoms Questionnaire; WCCL: Ways of Coping Checklist ; WPSI: Wahler Physical Symptoms Inventory

* Direct measure of psychosomatic symptoms.

### Study characteristics

Sixteen countries were represented in the eligible studies: the United States (*n* = 6), Germany (*n* = 4), Saudi Arabia (*n* = 3), China (*n* = 2), Malaysia (*n* = 2), Russia (*n* = 2) and one each from Nepal, India, Kazakhstan, Iran, Egypt, Nigeria, Tunisia, Spain, Italy, and Hungary (Table 1). In total, 27 medical schools and four teaching hospitals were represented. Sample sizes were reported in all studies and varied between *N* = 48 and *N* = 900 (mean = 330.19, median = 331; total *N* = 9,887). Twenty-two studies reported response rates, which ranged from 19.9 to 100%. The mean age of students in the 22 studies that reported it was 18–26.8. Self-identified sex was reported in 27 studies, and ranged between 3 and 75% female (excluding the study on menstrual symptoms which was 100% female-identifying).

Study design in the 29 studies included 24 studies that utilized a descriptive cross-sectional design, three longitudinal designs, and two quasi-experimental pre–post measures. Theoretical frameworks were reported in five studies: two studies utilized the transactional stress model [42,43], and one study each used the pituitary–adrenocortical regulation stress theory [44], the stress-diathesis theory [45] and the biopsychosocial model [46]. The remaining 24 studies did not report a theoretical framework.

### (Question 1) What is the prevalence of mental health-related somatic symptoms reported in the literature for medical students worldwide?

Twelve studies investigated the prevalence of mental health-related somatic symptoms. These percentages ranged from 5.7 [44] to 80.1% [47] and are summarized in Table 1. The weighted mean of all percentages is 26.3%.

### (Question 2) What are the types and characteristics of mental health-related somatic symptoms/disorders reported in medical students?

A number of authors did not use a direct measure of mental and physical health, but instead measured one or more physical factor(s), e.g. back pain, neck pain and then correlated them with one or more psychological factors, e.g. stress, depression (Table 1 for the physical and psychological variable(s) in each study).

Several studies evaluated musculoskeletal pain and found it associated with school-related depression (OR 2.93, 95% CI [1.73–4.98], *p* < .001) and other mental health-related somatic symptoms (OR 2.93, 95% CI [1.37–5.27], *p* = .004) [33]. Stress was correlated with low back pain as one facet of musculoskeletal pain (OR 2.9, 95% CI [1.4–5.9], *p* = .003) [29]. A history of depression was significantly correlated with musculoskeletal pain (OR = 2.95, 95% CI [1.54–5.64]) [55]. Being in the clinical years vs. the preclinical years was found to be associated with more musculoskeletal pain [34]; one study reported 2.9 greater odds of neck pain in clinical students, which increased to 3.5 greater odds if the pain was attributed to academic stress by the individual (*p* < .001) [36]; another found school-related depressive symptoms made it twice as likely for someone to report musculoskeletal pain [55].

Along with musculoskeletal pain, many other somatic symptoms were reported. Headache, tiredness and sleep disturbance were associated with academic burnout (all *ps* < .001) [59]. Frequency of stress was correlated with somatic symptoms (*r* = 0.71, *p* < .001), as was intensity of stress (*r* = 0.31, *p* < .01) [58]. Likely/definite anxiety was found to be an independent risk factor for functional gastrointestinal disorders, for example, irritable bowel syndrome (IBS) [30]. A likely or confirmed anxious state resulted in double the risk of having a functional gastrointestinal disorder (OR = 2.5, 95% CI [1.1–5.8] [30]. Suicidality was found to be associated with general pain and fatigue (OR 6.11, 95% CI [2.75–128.89], *p* = .003) [45]. Other general symptoms associated with mental distress were indigestion, altered bowel movements, stomach pain, fainting, dizziness, chest pain, shortness of breath, difficulty breathing, inability to take a deep breath, heart palpitations, tachycardia, sexual dysfunction, rapid urination, sleep disruption/insomnia, chronic fatigue, muscular tremors and tension headaches (Table 1).

There were no significant correlations between perceived stress and oligomenorrhea, amenorrhea or dysmenorrhea in the one article which assessed these in medical students (all *p* > .05) [49], though it is worth noting that research supports that stress contributes to menstrual disorders [62], making this a worthwhile investigation. Only one study found that the frequency of stress-related illnesses *decreased* after starting medical school; however, the data were derived from students’ recollections after approximately nine months to seven years of school [49]. This article compared MD, MD-PhD and PhD students, and although nothing was statistically significant in the MD student population, male-identifying MD-PhD students had significantly more colitis and hypertension, and female-identifying MD-PhD students significantly more gastritis and ulcers than the other groups; MD-PhD students reporting the highest stress levels out of the three groups [49]. None of the studies addressed psychological trauma or post-traumatic stress disorder.

Three studies included objective measures. One measured the rate of seroconversion after HBsAg (Hepatitis B) vaccination and found that medical students seroconverting after the first vaccine injection had significantly less anxiety than students who only seroconverted after further injections (*F* (l,44) = 6.28, *p* < 0.02) [38]. A separate examination of biomarkers in a group of medical students during a three-month intensive examination period, compared to a group not involved in exams, found significant decreases in cholesterol, high-density lipoproteins (HDL), and triglycerides, and significant increases in blood sugar, the ratio of low-density lipoproteins (LDL) to HDL and systolic blood pressure during the testing period in the exam group [44]. The authors attributed these changes to long-term stress, which can decrease cholesterol, triglycerides and cortisol, and hypothesized that compensatory processes in the body might then increase blood sugar and blood pressure [44]. The final study with objective measures assessed blood pressure, lipoprotein levels, body weight, smoking and family history of cardiovascular diseases and found that higher risk of cardiovascular disease as calculated from these variables was associated with higher levels of unexpressed anger and psychological distress [46].

Many of the studies compared the rates of mental health-related somatic symptoms among students identifying as female vs. male. Overall, studies found higher frequency of mental health-related somatic symptoms in self-identified females; for example, 30.4% of females vs. 14.9% of males [50] and 19.9% of female vs. 5.2% of males (*p* < .005) [61]. A third study found higher risk of mental health-related somatic symptoms in female individuals (*p* = .002) [55] along with higher frequency of headaches (*t* = 4.82, *p* < .01) [57]. Two weeks before an exam, females were more likely to have experienced mental health-related somatic symptoms (*p* < .0001) [56], and to have higher levels of autonomic symptoms compared to males (*r* = 0.46, *p* = .003); this was maintained at one day pre-exam (*r* = 0.901, *p* < .001) [56]. Four studies found no significant differences based on self-identified sex [32–34,52],and one study found that males had more physical symptoms of stress (AOR 1.5) [54].

### (Question 3) What are the different instruments used to measure mental health-related somatic symptomology among medical students?

Eleven different outcome measures were used to assess mental health-related somatic symptoms, as described in Table 2 [63–69,71–73]. The most commonly used, by four studies, was the Patient Health Questionnaire-15 (PHQ-15). Three studies used the Revised Symptom Checklist (SCL-90-R). Each of the eight remaining studies used a different measure, and five studies used self-developed questionnaires. See Table 2 for the validity and reliability of each outcome measure, when available.

**Table 2.** Validity & reliability of mental health-related somatic symptom outcome measures.

Mental health-related somatic outcome measure	Validity	Reliability
Enugu Somatization Scale (ESS)	Intrinsic validity: 0.9654 [54]	Cronbach’s *α*: 0.932, internal consistency: 0.936 [54]
Framingham Type A Behavior Pattern Scale (FTAS)	*Not found*	Cronbach’s α: 0.68 [63]
Patient Health Questionnaire-15 (PHQ-15)	PHQ-9: sensitivity 89% and specificity 97% in college students [64]; GAD-7: sensitivity 73.3%, specificity 67.3%; PHQ-15: sensitivity 78%, specificity 71% (for DSM-IV somatoform disorder diagnosis) [65]	PHQ-9 Cronbach’s α: 0.88; GAD-7 Cronbach’s α: 0.89; PHQ-15 Cronbach’s *α*: 0.82 [66]
Psychosomatic Problems Scale (PPS)	Multidimensionality: 0.80% at 0.01 alpha [67]	Person Separation Index: 0.83 [67]
Symptoms Checklist 90 – Revised (SCL-90-R)	Comparative Fit Index: 0.967–0.995 [68]	Cronbach’s *α*: 0.89–0.93 [68]
Screening for Somatoform Disorders (SOMS-2)	Sensitivity: 86.0%; Specificity: 95.5% [69]	Cronbach’s *α*: 0.83 [69]
Self-Reporting Questionnaire-20 (SRQ-20)	Sensitivity: 63.0%	
Specificity: 88.0% [70]	Cronbach’s *α*: 0.84 [71]	
Somatic Symptom Scale-8 (SSS-8)	Mental health-related somatic symptoms were correlated with anxiety (*r* = 0.55 [95% CI, 0.52–0.58]), depression (*r* = 0.57 [95% CI, 0.54 to 0.60]), health (*r* = −0.24 [95% CI, –0.28 to −0.20]), and health care utilization (incidence rate ratio, 1.12 [95% CI, 1.10 to 1.14]) [6^9^]	Cronbach’s *α*: 0.81 [72]
Veins Autonomic Symptoms Questionnaire (VASQ)	Not found	Not found
Wahler Physical Symptoms Inventory (WPSI)	*F =* 48.75 (*p* < .001) difference in means [73]	Test-retest reliability: 0.81 [73]
Diagnostic criteria for somatoform autonomic dysfunction (SAD)	Prevalence of somatization: 0.8–5.9% with strict DSM/ICD criteria application. Estimates higher with less strict application [74]	Diagnoses from the DSM vs. ICD varied significantly. Data from questionnaire vs. clinical interviews also varied [74]

#### Psychological symptoms

The studies that collected separate psychological measures (e.g. stress, depression, anxiety, burnout) to correlate with physical symptom reports, instead of one measure mental and physical symptoms, included 12 separate measures, as seen in Table 1.

#### Physical symptoms

Seven separate measures of physical symptoms were used (to correlate with psychological symptom reports, instead of one measure of mental and physical symptoms) also in Table 1. In the three studies with physiological as opposed to self-reported assessments, authors measured speed and timing of seroconversion after vaccine [38]; blood levels of leukocytes [44], eosinophil granulocytes [44], lymphocytes [44] and cortisol [44]; cholesterol [44,46]; HDL, LDL, LDL/HDL quotient [44]; triglycerides [44,46]; blood sugar [44]; blood pressure [44,46]; body weight [46]; reaction time and number of false reactions [44] and maximal muscle ergometry [44].

### (Question 4) is there support for any approaches to predict, prevent, or treat mental health-related somatic symptoms in medical students?

Five studies assessed factors that have the potential to be helpful in predicting, preventing or treating mental health-related somatic symptoms in medical students; one of these performed an intervention. The four non-interventional factors were resilience [37], social support [38], coping strategies [58] and trait mindfulness [48]; the interventional study [43] also assessed mindfulness, and was based on Jon Kabat-Zinn’s Mindfulness-Based Stress Reduction (MBSR) program [75,76]. Resilience was measured with a binary ‘yes/no’ scale on dimensions of resiliency as conceptualized by the authors: use of relaxation techniques, satisfaction with academic studies, religiosity, eating habits, physical activity, musical instrument practice and emotional/social support [37]. High scores on these dimensions of resiliency were found to be significantly correlated with the SOMS-2 somatization index, such that students with higher resiliency had significantly lower somatization (*r*_s_ = −0.207, *p* < .001) [37]. In the next study, social support was evaluated with the Interpersonal Support Evaluation List (ISEL) that measures four types of social support: appraisal, belonging, tangible and self-esteem [38]. Higher levels of self-reported social support were found to be significantly associated with better immune responses to the Hepatitis B vaccine (*t* = 2.21, *p* < .05, *df* = 34) [38]. In the third study, coping strategies were assessed with Coping Strategies Inventory (CSI) [58], which classifies coping strategies as either an Engagement or Disengagement. Engagement includes the strategies of problem-solving, cognitive restructuring, seeking social support and expressing emotions, while disengagement is regarded as less helpful, and includes problem-avoidance, wishful thinking, social withdrawal and self-criticism [58]. The study found that self-reported use of Engagement coping strategies were negatively associated, and Disengagement positively associated, with depression; in addition, wishful thinking was correlated to higher somatic complaints (partial correlation = 0.34, *p* < .01) [58]. The fourth study evaluated the mediating effect of trait mindfulness on psychosomatic burden, stress, anxiety and sleep quality; trait mindfulness was assessed with the Five Facets Mindfulness Questionnaire (FFMQ) at the beginning and end of the first semester of the second year of medical school [48]. Certain facets of trait mindfulness, specifically ‘nonjudgement of inner experiences’ and ‘acting with awareness’ were negatively correlated with anxiety, stress, psychosomatic symptoms and poor sleep quality, and ‘acting with awareness’ was positively correlated, though these correlations were not statistically significant [48].

The intervention consisted of a mindfulness program delivered in eight, 2-h sessions over 16 weeks, and was offered to second through fifth-year medical students in a medical program in Spain [43]. The sessions included exposure to cognitive therapies, attention exercises, body-scanning and meditation, among other topics [75,76]. The results were positive, showing significantly decreased stress (*F* (1, 141) = 8.23, *p* = .005) and physical symptoms of stress (*F* (1, 141) = 6.22, *p* = .014) compared to a control group [43].

#### Bias

All of the studies in this review except for one [43] are single-group observational design; therefore, most findings are based on lower levels of evidence and subject to risk of bias due to a lack of comparison groups. In addition, risk of bias may exist among studies with longer durations (i.e. risk of response), and studies with incomplete representation of the entire population of interest (e.g. the study aim was to assess all medical students but not every student year was sampled from) [29,35,36,45,47,48]. Selection bias is highly likely with all studies being voluntary, as is response bias with the almost ubiquitous use of self-reported outcome measures. Publication bias is likely due to our use of only published works. We also observed lower response rates (below 60%) [32,33,49,57] and underpowered sample sizes.

## Discussion

This scoping review is the first of its kind to assess physical symptoms of stress in medical students, and finds varying degrees of the prevalence and type of symptoms, largely due to the range of research questions in the original articles and differing outcome measures. This review also reveals a dearth of practical information on predicting, preventing or treating physical symptoms of stress in medical students – an area ripe for further investigation. Although there exists considerable research on how personality traits and coping strategies influence medical students’ stress levels and mental health, further research on personality and coping as they relate to physical symptoms of stress would be beneficial for understanding what may predispose medical students to experiencing physical symptoms – given that both mental and physical symptoms can lead to reduced quality of life [10–12] and occupational attrition [8,9].

The social, economic and cultural characteristics of the student samples, which were drawn from schools in 16 different countries, could play a significant role in how medical students experience or report mental health-related somatic symptoms. For instance, in the United States, medical school is a four-year graduate program after four years of undergraduate schooling, whereas in other countries an undergraduate degree is not required, making the average age of matriculants lower internationally [70]. During schooling, when stress levels are rising, age may be influential on the experience of stress and how developed a student’s coping strategies are, with evidence that older students have more adaptive coping strategies, such as better ability to plan [77]. We compared the prevalence of any type of mental health-related somatic symptomology between international and U.S.-based samples, which did not show a statistically significant difference (*n* = 12, *r* = –0.276, *p* = .386), though there was only one U.S. study reporting prevalence which means this statistic is unreliable. Mean age was likewise not significantly correlated with prevalence of any mental health-related somatic symptoms (*n* = 9, *r* = –0.202, *p* = .602); nor was the sample being in preclinical vs. clinical years (*n* = 6, *r* = –0.349, *p* = .498). We also questioned if the experience or reporting of mental health-related somatic symptomology has changed over time, as the articles which reported prevalence spanned 28 years, but found no pattern to the year which the study was published and the prevalence of symptoms (*n* = 9, *r* = –0.198, *p* = .610).

Differences in reported gender experiences of physical symptoms of stress were mixed, but appear to suggest that female medical students may experience or report more physical symptoms. Previous research on gender differences indicates that female-identifying students typically report higher stress levels [78], as well as more emotion-focused coping [79]. This conceptualization of coping describes emotion-focused coping as occurring when an individual attempts to suppress or change their emotions about a situation or concern, as opposed to problem-focused coping which occurs when one is more actively exploring solutions [80]. Problem-focused coping typically results in lower stress levels because a concrete action has been taken [80]. Although coping strategies’ potential association with physical symptoms of stress has not been investigated, better coping strategies lead to lower perceived stress, and therefore may also lead to lower physical symptoms of stress.

Eleven different outcome measures were used for exploring physical symptoms of stress, including two for which no other literature could be found (Table 2). There were also 12 different self-reported psychological measures, seven self-reported physical measures and 17 objective measures present within the 29 studies, demonstrating overall inconsistency in how symptoms were assessed and measured. The variety in instruments could certainly lead to differing estimates of prevalence. Of the four studies which used the PHQ-15, three reported prevalence, which ranged from 5.7 to 22.4%. The two studies which used the SCL-90 and collected prevalence of mental health-related somatic symptoms report 21.7 and 24.5%. The values from the PHQ-15 and SCL-90 clearly do not span the wide range present when considering all the measures, but with only a few values it’s impossible to determine if that is due to scale consistency or chance. A comparison of reliability as measured by Cronbach’s alpha suggests that the Enugu Somatization Scale (ESS; *α* = 0.932 [63]) and the SCL-90-R (*α* = 0.89–0.93 [69]) had the highest reliability in their respective samples, and the Framingham Type A Behavior Pattern Scale had the lowest (FTAS; *α* = 0.68 [64]). The ESS also had high validity (intrinsic validity = 0.954 [63]), as did the SCL-90-R (Comparative Fit Index = 0.967–0.995 [69]]; the specificity of the Screening for Somatoform Disorders (SOMS-2) was also comparatively high, at 95.5% [71]. (Table 2 for all data). These data suggest the ESS and the SCL-90-R may be the most valid and reliable for measuring mental health-related somatic symptoms in medical students, but clearly further research would be beneficial.

Interventions for mental health issues in medical students are becoming more common, with schools initiating curricular change to add, for example, mindfulness activities or electives on stress-reduction [81]. Interventions addressing physical symptoms of stress have not, as far as we know, been suggested or trialed for medical students as a group. In the general population, treatment for mental health-related somatic symptoms/somatic symptom disorders is typically multidimensional, with cognitive behavioral therapy aimed at reducing illness behaviours and catastrophizing of physical symptoms, techniques aims at regulating the neuroendocrine system [82] and the use of antidepressant medication [74]. Functional relaxation also has evidence to support its use [83]. Medical students in the United States typically have free resources for counseling, but may not be able to regularly utilize them due to time constraints and stigma; in addition, academic programs usually only cover 4–6 therapy sessions. International students may or may not have access to counseling. Therefore, the addition of similar or potentially beneficial content in group or asynchronous settings may be much more feasible.

### Limitations

Noteworthy limitations of this review include that the majority of the studies drew samples from only one medical school, reducing generalizability; the observational cross-sectional design also limits the ability to assess incidence of mental health-related somatic symptoms. The outcome measures were largely self-reported which increases response bias. By including only peer-reviewed literature, publication bias is possible. None of the studies collected data on persons with marginalized gender identities or sexual orientations, even though membership in a marginalized group has been shown to lead to significantly higher stress levels which could in turn lead to higher rates of mental health-related somatic symptoms [84,85].

### Implications and recommendations

Interventions teaching active coping strategies, like positive reframing, have been found to be beneficial [86,87], as has social support [88], regular exercise [89] and good sleep habits [90]. Medical schools that have implemented mindfulness, yoga and stress-management education as an elective course or as mandatory curricula have promising results [91–100], yet medical students may be given good advice but not feel they have time to implement new techniques. Further research is needed to determine which interventions are effective in reducing mental health-related somatic symptoms in medical students, along with studies to determine acceptability and ease of incorporation into the busy daily life of a medical student.

Looking beyond schooling and assessing the impact of mental health-related somatic symptoms on physician and healthcare workers’ career, trajectories and patient outcomes would be valuable, particularly given the known shortages in the healthcare workforce that are expected to progressively worsen [6,7]. It is currently unknown if the changes in responsibilities and autonomy that come with promotion from student to professional alter mental health-related somatic symptom expression or risk of manifesting physical disease. Further investigation into biomarkers associated with stress (e.g. cortisol), which give insight into the physiological sequelae of emotions, is also warranted in this population.

## Conclusion

This scoping review found that medical students frequently report mental health-related somatic symptoms, likely with a great deal of unstudied symptomology, and unknown downstream effects. Further research is vital to assess prevalence, severity, protective factors, sequelae and therapeutic interventions to assess and treat mental health-related somatic symptoms in medical students, potentially reducing occupational attrition and economic costs, and enhancing the quality of life and career longevity of our healthcare professionals.

## Data Availability

Data sharing is not applicable to this article as no new data were created or analyzed in this study.
